# One-step chemical vapor deposition synthesis and supercapacitor performance of nitrogen-doped porous carbon–carbon nanotube hybrids

**DOI:** 10.3762/bjnano.8.267

**Published:** 2017-12-12

**Authors:** Egor V Lobiak, Lyubov G Bulusheva, Ekaterina O Fedorovskaya, Yury V Shubin, Pavel E Plyusnin, Pierre Lonchambon, Boris V Senkovskiy, Zinfer R Ismagilov, Emmanuel Flahaut, Alexander V Okotrub

**Affiliations:** 1 Nikolaev Institute of Inorganic Chemistry, SB RAS, 630090 Novosibirsk, Russia; 2 Novosibirsk State University, 630090 Novosibirsk, Russia; 3 CNRS, Institut Carnot Cirimat, F-31062 Toulouse, France; 4 St. Petersburg State University, 7-9, Universitetskaya Nab., St. Petersburg 199034, Russia,; 5 II Physikalisches Institut, Universität zu Köln, Zülpicher Straße 77, 50937 Köln, Germany; 6 Boreskov Institute of Catalysis, SB RAS, 630090 Novosibirsk, Russia,; 7 Institute of Coal Chemistry and Materials Science FRC CCC SB RAS, Kemerovo 650000, Russia

**Keywords:** bimetallic catalyst, electrochemical impedance spectroscopy, N-doped carbon, porous carbon–carbon nanotube hybrid, supercapacitor

## Abstract

Novel nitrogen-doped carbon hybrid materials consisting of multiwalled nanotubes and porous graphitic layers have been produced by chemical vapor deposition over magnesium-oxide-supported metal catalysts. CN*_x_* nanotubes were grown on Co/Mo, Ni/Mo, or Fe/Mo alloy nanoparticles, and MgO grains served as a template for the porous carbon. The simultaneous formation of morphologically different carbon structures was due to the slow activation of catalysts for the nanotube growth in a carbon-containing gas environment. An analysis of the obtained products by means of transmission electron microscopy, thermogravimetry and X-ray photoelectron spectroscopy methods revealed that the catalyst's composition influences the nanotube/porous carbon ratio and concentration of incorporated nitrogen. The hybrid materials were tested as electrodes in a 1M H_2_SO_4_ electrolyte and the best performance was found for a nitrogen-enriched material produced using the Fe/Mo catalyst. From the electrochemical impedance spectroscopy data, it was concluded that the nitrogen doping reduces the resistance at the carbon surface/electrolyte interface and the nanotubes permeating the porous carbon provide fast charge transport in the cell.

## Introduction

A combination of multiple nanostructured materials in one hybrid systems often allows a synergistic improvement of the properties of the individual components. This is important for many practical fields and particularly for electrochemical production of energy [1], where the electrodes should possess a high surface area available for electrolyte ions, good wettability and electrical conductivity. Carbon is a traditional material for electrochemical applications owing to its mechanical and chemical stability, light weight, as well as the possibility to control the properties depending on the morphology and chemical bonding organization [2]. Porous graphitic materials like activated carbon may have a specific surface area exceeding 3000 m^2^ g^−1^ [3], while in this case they lose the packing density and conductivity. The latter characteristics can be improved by incorporation of carbon nanotubes (CNTs) or fibers in an electrode material [4–5].

A typical synthesis procedure for carbon–carbon hybrid materials includes the mechanical mixing of the components, previously synthesized separately by different methods (ex situ synthesis). The mixing is usually carried out in a solvent, but since carbon materials are hydrophobic, they often should first be oxidized. In this case the hybridization degree (bonding, interconnection, dispersion) is strongly dependent on the choice of nanocarbon materials and surface functionalities, which has been evidenced by electrochemical investigation of ex situ prepared hybrid materials [6–9]. Another less common strategy consists of CNT growth by catalytic chemical vapor deposition (CCVD) over catalyst nanoparticles predeposited on the graphitic surfaces [10–13]. The obtained hybrids are characterized by tight bonding between the components, which is necessary for fast charge transfer in an electrochemical cell. However, in this case, the components are connected only through this limited interface that may not allow the synergism from their hybridization to be fully reach.

The synthesis of two morphologically different carbon nanomaterials with a "one pot" strategy seems the most practical way for the formation of the hybrid nanomaterial. The advantages of such a method are the reduction in the number of production steps and a natural and more homogeneous interconnection of the components. To the best of our knowledge, there are only few works devoted to one-step formation of porous carbon–CNT hybrids for energy storage applications. Lei et al. have reported the CCVD synthesis of nitrogen-doped ordered mesoporous carbon and multiwalled CNTs (MWCNTs) with the use of a silica SBA-15 template impregnated by iron nitrate [14]. Electrochemical tests of the obtained materials in 6 M KOH electrolyte evidenced a significant reduction of the interfacial contact resistance of the electrode with the insertion of MWCNTs. Luo et al. have prepared a hierarchical porous carbon–MWCNT hybrid by carbonization of a mixture of phenolic resin and nickel hydroxide in hydrogen atmosphere [15]. The hybrid showed improved cycling stability in lithium–sulfur batteries as compared to the electrode made from porous carbon only. Cai et al. have synthesized N-doped hierarchical porous carbon–CNT hybrids using a melamine-formaldehyde resin, Fe/Co catalyst, and nanostructured CaCO_3_ as a template [16]. The electrodes from the synthesis products exhibited a good reversible capacity and long-term cycling stability. The role of CNTs in such hybrids was to enhance the electrical conductivity and to act as a physical barrier, blocking large pores in the second carbon component.

Magnesium oxide (MgO) is a suitable substrate for the synthesis of porous carbon–CNT hybrids owing its high surface area, chemical and thermal stability under growth conditions, and easy removal from carbon products using a diluted hydrochloric acid aqueous solution. Nanoporous carbon has been shown to form because of the carbonization of the carbon source at the surface of MgO [17]. Advantages of this method are that carbon stabilization and activation steps are not required, as well as the control of the size and the volume of the pores through the design/choice of the used MgO particles. The nucleation and growth of CNTs typically require stabilization of the catalytic nanoparticles at elevated temperatures, and MgO is often used for this purpose [18]. Rümmeli et al. have shown that under typical CCVD conditions for CNT growth and in the absence of a deposited catalyst, the MgO nanoparticles are coated by few layer graphene-like material [19]. Dervishi et al. have synthesized nanoscale graphene structures or MWCNTs by varying the active catalyst loading in a Fe/Mo/MgO system using radio-frequency CCVD [20].

In the present work, we demonstrate a simultaneous CCVD synthesis of MWCNTs and porous carbon on the surface of MgO impregnated with bimetallic (transition metal/molybdenum) catalyst precursors. Acetonitrile was added to the methane feedstock to incorporate nitrogen into the graphitic network, which is beneficial for the electrochemical performance of the carbon materials [21]. With the purpose to study the effect of the nature of the transition metal on the nitrogen content and the structure of the porous carbon–MWCNT hybrid, we used cluster-type polyoxomolybdates of Co, Ni, and Fe as catalyst precursors. In earlier work, we have shown that the thermal decomposition of such clusters produces metal molybdates which, when heated in hydrogen atmosphere, are reduced to bimetallic alloys catalyzing the CCVD growth of few-walled CNTs [22]. Here, the catalysts were activated by slow heating in a carbon-containing environment from room temperature to 900 °C. The structure and composition of the obtained CN*_x_* hybrids were correlated with the data of cyclic voltammetry (CV) and electrochemical impedance spectroscopy (EIS) measurements in 1 M H_2_SO_4_ electrolyte.

## Experimental

### Synthesis

Catalysts were prepared using polyoxomolybdate clusters of the ε-Keggin-type structure Mo_12_O_28_(μ_2_-OH)_12_{Ni(H_2_O)_3_}_4_ and Mo_12_O_28_(μ_2_-OH)_12_{Co(H_2_O)_3_}_4_ and the Keplerate-type structure [Н_4_Мо_72_Fе_30_O_254_(СН_3_СОО)_10_{Мо_2_O_7_(Н_2_O))(Н_2_Мо_2_O_8_(Н_2_O)}_3_(Н_2_O)_87_] as precursors. The compounds were synthesized following the procedures described elsewhere [23–24]. An aqueous suspension of a compound was stirred with MgO hydrosol at 60 °C and after water evaporation the precipitate was dried at 80 °C overnight. This resulted in cream-colored, orange–peach, and very light orange color in the case of {Ni_4_Mo_12_}, {Co_4_Mo_12_}, and {Fe_30_Mo_72_}, respectively. According to the atomic emission spectral analysis (iCAP-6500 spectrometer), the loading of Ni, Co, or Fe on the MgO substrate was about 1 wt %.

**Figure 1:**
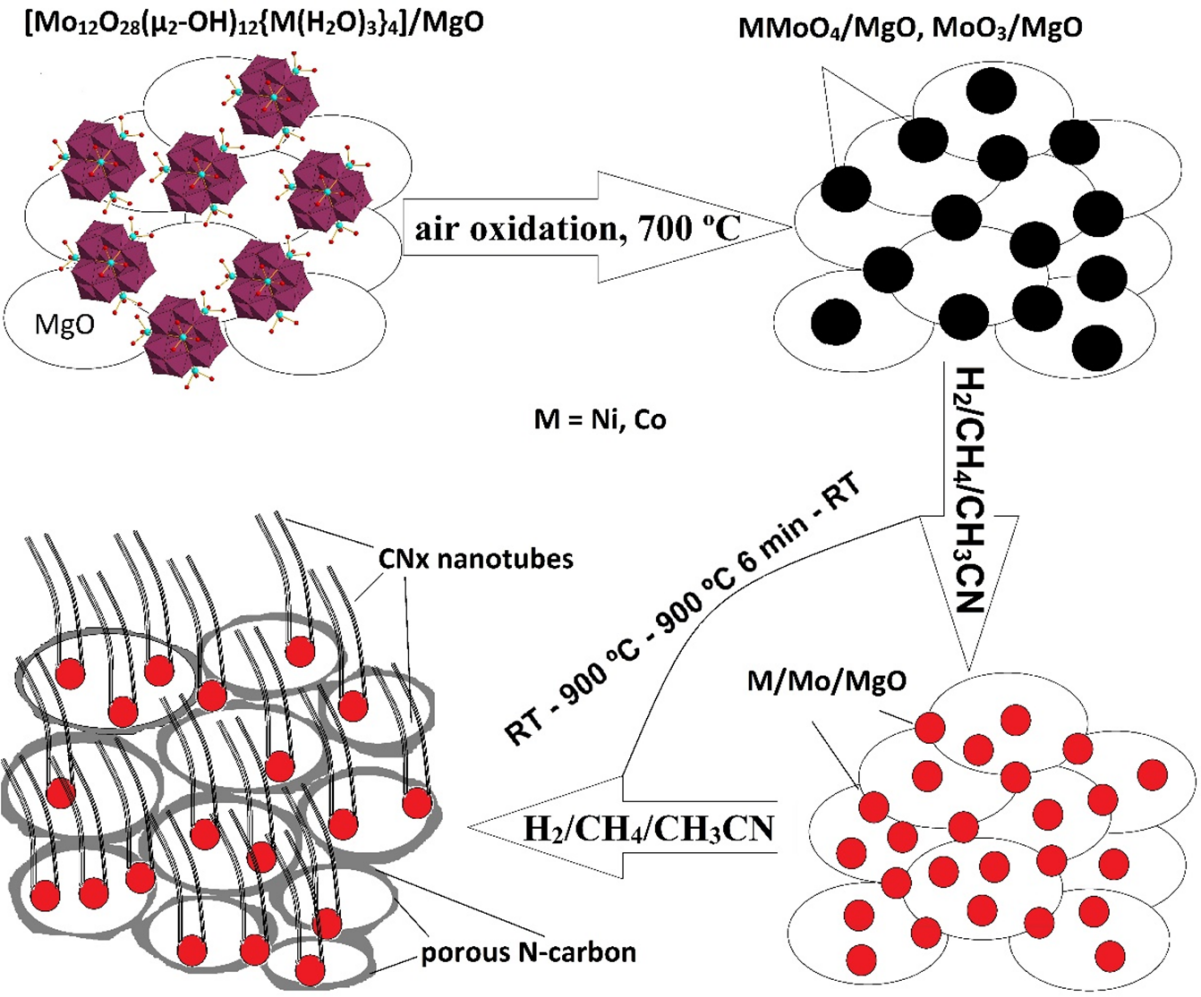
Schematic representation of nitrogen-doped porous carbon–carbon nanotube hybrid formation with the use of Ni or Co polyoxomolybdate clusters.

CN*_x_* hybrid materials were synthesized in a horizontal tubular reactor 1.6 m in length and 7 cm in diameter with a constant temperature zone in the center of ≈15 cm. Before the synthesis, powders of {Ni_4_Mo_12_}/MgO, {Co_4_Mo_12_}/MgO, and {Fe_30_Mo_72_}/MgO (≈60 mg each) were calcined in air atmosphere in a muffle furnace at 700 °C for 10 min. After that, the colors of the powders all turned to grey. The obtained products were uniformly distributed into ceramic boats, which were placed in the center of the reactor. The mixture of CH_4_ (67.5 mL/min) and H_2_ (307.5 mL/min) gases was bubbled through acetonitrile prior to circulation in the reactor. Simultaneously, the reactor was heated at 5 °C/min. After reaching 900 °C, the temperature was kept constant for 6 min and then naturally decreased to room temperature. At 120 °C, the reactor was flushed with N_2_ for 30 min. The MgO support and all accessible (not carbon-coated) metals were eliminated from the products using an overnight treatment by a diluted aqueous hydrochloric acid solution. A general synthesis scheme is proposed in Figure 1.

### Instrumental methods

The simultaneous thermal analysis of the synthesized samples was carried out on a STA 449 F1 Jupiter^®^ thermal analyzer (NETZSCH) in an O_2_ (10 mL/min)/Ar (40 mL/min) atmosphere. The sample weight was about 10 mg and the heating rate was 10 °C/min. Nitrogen adsorption experiments were conducted at 77 K on an ASAP 2400 (Micromeritics) instrument. The total surface area was calculated using the Brunauer–Emmett–Teller (BET) equation on the basis of adsorption data in the partial pressure (*P*/*P*_0_) range of 10^−5^–1.0. X-ray diffraction (XRD) patterns were recorded on a SHIMADZU XRD-700 powder diffractometer using Cu Kα radiation. Scanning electron microscopy (SEM) and transmission electron microscopy (TEM) images were obtained on a JEOL JSM-6700F microscope and a FEI Tecnai-F20 microscope, respectively. Raman spectra were measured on a Triplemate spectrometer using the 488 nm radiation from an Ar^+^ laser. The nitrogen state in the samples was studied by means of X-ray photoelectron spectroscopy (XPS) and near-edge X-ray absorption fine structure (NEXAFS) at the facility of the Russian-German beamline at the Berliner Elektronenspeicherring für Synchrotronstrahlung (BESSY II) station. XPS spectra were measured at an energy of monochromatized radiation equal to 830 eV. NEXAFS spectra were acquired in the total-electron yield (TEY) mode with the monochromatized incident radiation of ≈0.08 eV for the carbon region and ≈0.17 eV for the nitrogen region. The spectra were normalized to the primary photon current from a gold-covered grid recorded simultaneously.

### Electrochemical measurements

The electrochemical properties of the samples were studied at room temperature in a three-electrode flat cell in a similar manner as described before [25]. A sample (5 mg) was dispersed in 1 mL of ethanol containing 1 μL of Teflon F-4D aqueous suspension (62%) as a binder. The mixture was prepared using a mortar and pestle, before being rolled out into a 40 µm film, which was dried at room temperature. An Ag/AgCl electrode filled with a saturated KCl aqueous solution was used as the reference electrode and Pt foil was used as the counter electrode and the current collector for the working electrode. The working and counter electrodes were separated by a nonwoven 20 μm thick polypropylene cloth, which was impregnated with an excess of 1 M H_2_SO_4_ electrolyte. Cyclic voltammetry (CV) curves were recorded on a Elins P–30s potentiostat. The potential ranged from 0 to +1 V with a scan rate between 2 and 1000 mV s^−1^. The specific capacitance (C) of an electrode was determined with the formula *C* = *A* / (*V*_s_ × *m*), where *A* is the square of the positive curve, *V*_s_ is the scan rate and *m* is the mass of carbon nanomaterial. EIS measurements were performed on a SP-300 potentiostat/galvanostat (Bio-Logic Science Instrument, France) in the frequency range of 20 kHz–10 mHz at zero potential with an amplitude of 10 mV.

## Results and Discussion

### Material structure

**Figure 2:**
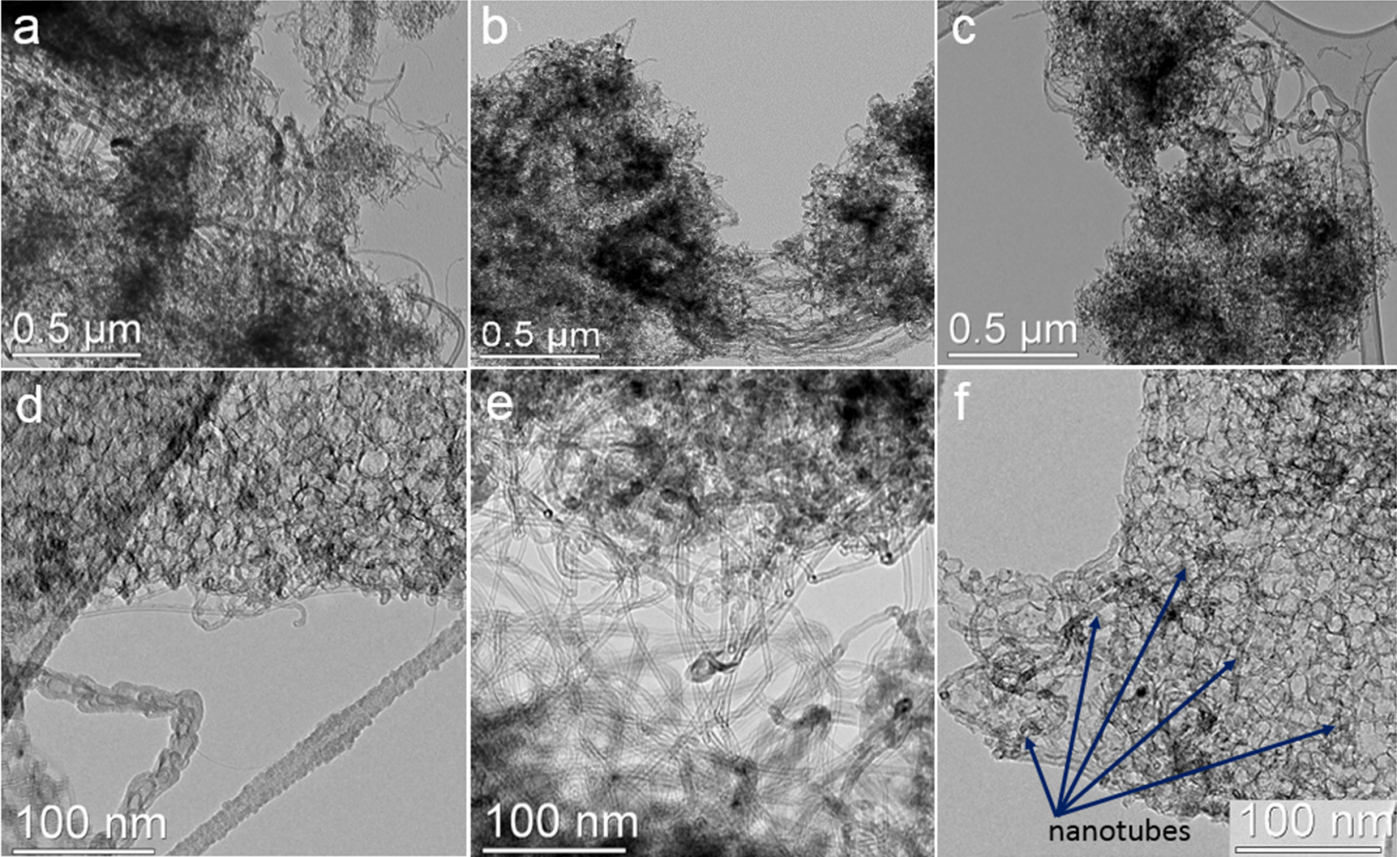
Low- and high-magnification TEM images of CN*_x_* samples synthesized using Co/Mo (a, d), Ni/Mo (b, e) and Fe/Mo (c, f) catalysts. The arrows in panel (f) indicate nanotubes that permeate the porous carbon.

The SEM analysis of the samples, obtained after dissolution of the MgO support, evidenced the co-existence of CNTs and nontubular carbon (Figure S1 in Supporting Information File 1). TEM images revealed that the nontubular carbon is highly porous with a pore size between 8–20 nm (Figure 2). High-resolution TEM images demonstrated the layered structure of these carbons (Figure S2 in Supporting Information File 1). Since the average pore size determined from the N_2_ sorption measurements was ≈12 nm for all samples, we conclude that this carbon material was templated by MgO nanoparticles. The nanotubes permeate the porous carbon (Figure 2a,c,f) and connect the flakes (Figure 2b). The supported metals catalyzed the growth of the MWCNTs with different diameters and morphologies (see, for example, Figure 2d), depending on the nature of the catalytic nanoparticles and possible different wetting behavior toward MgO. According to the statistical analysis of the TEM images, the average outer diameter of the MWCNTs is 5 nm, 10 nm and 16 nm for Ni/Mo, Co/Mo and Fe/Mo catalysts (see histograms in Figure S3 in Supporting Information File 1), respectively.

The NEXAFS C K-edge spectra of the samples exhibited an intense π* resonance and a split σ* resonance (Figure S4 in Supporting Information File 1), which indicate the preferable sp^2^-hybridization of carbon atoms and their good structural organization in the layers of MWCNT–porous carbon.

**Figure 3:**
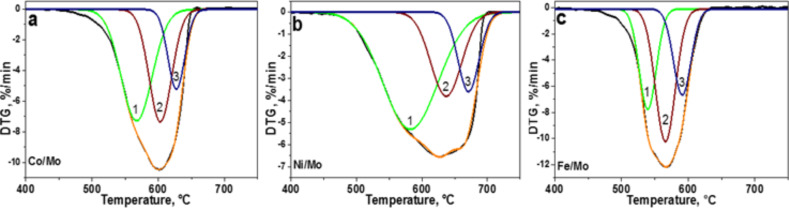
Differential thermogravimetric (DTG) curves of CN*_x_* materials synthesized using Co/Mo (a), Ni/Mo (b) and Fe/Mo (c) catalysts. The curves have been fitted by three components, assigned to different carbon species.

In order to evaluate the quantity of porous carbon and MWCNTs in the samples, we performed thermogravimetric (TG) analysis under synthetic air atmosphere (Figure 3 and Figure S5 in Supporting Information File 1). The samples started to lose a weight at 440–450 °C and finished at 640, 690, and 620 °С, for the catalysts Co/Mo, Ni/Mo, and Fe/Mo, respectively (Figure S5 in Supporting Information File 1). The residual mass of ≈5% corresponds to metal oxides. The broad peaks in the differential scanning calorimetry (DSC) curves are indicative of a variety of carbon nanostructures. In the case of the sample produced using the Fe/Mo catalyst, the DSC curve showed two distinct exothermic peaks at 540 and 570 °С (Figure S5c in Supporting Information File 1), which could be related to the combustion of porous carbon and MWCNTs, respectively.

Asymmetry in the differential thermogravimetric (DTG) curves (Figure 3) confirms the synthesis of carbon species of different thermal stability [26]. The curves have been fitted by three components, which were respectively assigned to the combustion of porous carbon (component 1), MWCNTs with many defects like the nanofibers shown in Figure 2d (component 2), and finally, more structurally perfect MWCNTs (component 3). The ratios of the components and their peak temperatures are collected in Table S1 in Supporting Information File 1. The DTG temperature is dependent on the catalyst composition and for all components it grows in a sequence Fe < Co < Ni. The temperature of the maximal rate loss for porous carbon is 539–580 °С (component 1), which is characteristic for mesoporous carbon with a relatively low nitrogen content of 0.5–1.0 wt % [27]. The component 2 is observed between 566 and 640 °С and the values agree well with the oxidation temperature of nitrogen-doped MWCNTs produced at 900 °С [28]. The temperature of the DTG component 3 varies from 591 °С (Fe/Mo catalyst) to 670 °С (Ni/Mo catalyst). A comparison of the integral normalized ratios of the DTG components (Table S1 in Supporting Information File 1) indicates that the largest fractions of porous carbon and highly defective MWCNTs were obtained in the materials produced using the Ni/Mo catalyst and Fe/Mo catalyst, respectively.

**Figure 4:**
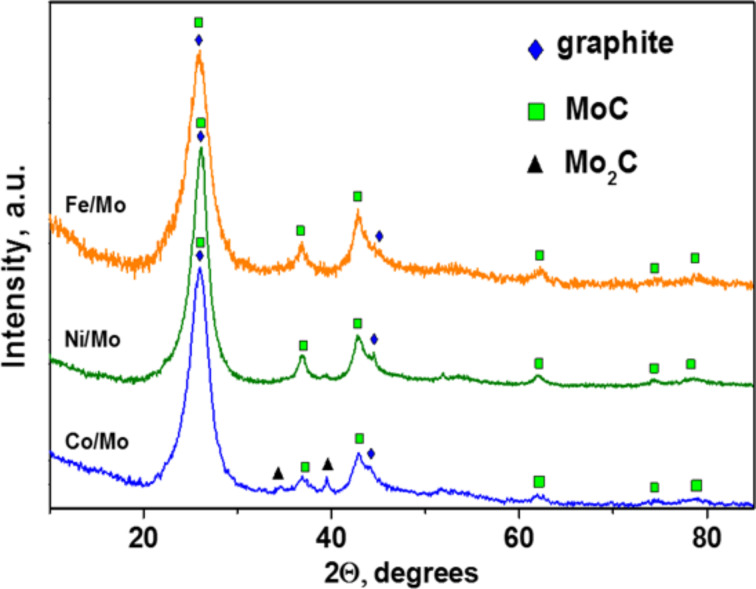
X-ray diffraction patterns of CN*_x_* materials synthesized using Co/Mo, Ni/Mo and Fe/Mo catalysts.

XRD patterns of the samples exhibited three main phases. One of them is a carbon phase with reflections from the (002) and (101) graphitic planes at 2θ ≈ 26° and 44.3°. These reflections overlap with the reflections corresponding to cubic modification of Mo_2_C (PDF2 card 000-89-2868). The third phase is cubic modification of MoC (PDF2 card 000-89-2868) (Figure 4). It is difficult to detect individual metallic phases such as Co, Ni, and Fe apparently due to the small amount present. Furthermore, these metals have a lower linear absorption coefficient in comparison with heavier Mo. Actually, the XRD analysis of the decomposition products of {Ni_4_Mo_12_}, {Co_4_Mo_12_}, and {Fe_30_Mo_72_} cluster compounds in air have revealed the presence of the phases NiMoO_4_ and CoMoO_4_ [22] as well as Fe_2_(Mo_4_)_3_ [29]. The reduction of these oxides in a CH_4_/H_2_ flow at 900 °C yields bimetallic particles as has been shown in our previous investigation [22]. Since these nanoparticles are located at the tips of the nanotubes, they correspond to encapsulated catalytic nanoparticles which allowed the growth of the MWCNTs.

### Electronic state of nitrogen in hybrid materials

The nature of the electronic state of nitrogen in the synthesized materials was investigated by XPS and NEXAFS spectroscopy. The nitrogen concentration was determined from the ratio of the area under the C 1s and N 1s peaks taking into consideration the photoionization cross-sections for elements at the given photon energy. The values derived from the survey XPS spectra of the samples synthesized using Ni/Mo, Co/Mo, and Fe/Mo catalysts are 0.9, 1.5, and 2.3 atom %, respectively. The obtained concentrations correlate with nitrogen solubility in liquid nickel and binary nickel-containing alloys [30].

**Figure 5:**
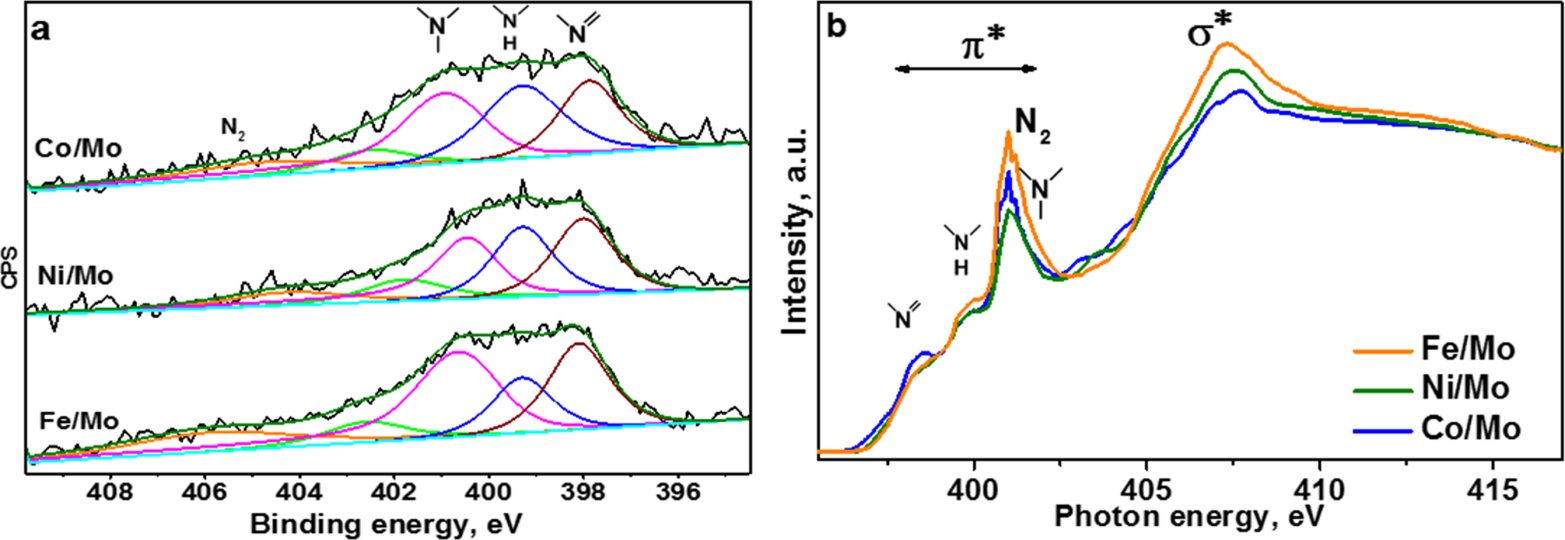
X-ray photoelectron spectroscopy (XPS) N 1s spectra (a) and near-edge X-ray absorption fine structure (NEXAFS) N K-edge total-electron yield (TEY) spectra (b) of CN*_x_* materials synthesized using Fe/Mo, Ni/Mo, and Co/Mo catalysts.

The XPS N 1s spectra of the three hybrid materials are compared in Figure 5a. The spectra were fitted by five components corresponding to different chemical forms of nitrogen. The components at ≈398.0, ≈399.3, and ≈400.7 eV are commonly assigned to so-called pyridinic, pyrrolic, and graphitic nitrogen configurations [31]. The next energy component located in our spectra within 401.8–402.7 eV is assigned to oxidized nitrogen configurations and clustered nitrogen substitutions. The highest energy component is attributed to nitrogen molecules located inside the MWCNTs [32]. The ratio between the different forms of nitrogen in the hybrid materials depends on the composition of the metal catalyst (Table S2 in Supporting Information File 1). The main form in all samples is graphitic nitrogen, which directly substitutes the sp^2^-hybridized carbon atom. A ability to insert this nitrogen in the carbon network follows the order Ni < Co < Fe. The material synthesized using the Ni/Mo catalyst contains equal fractions of pyridinic nitrogen and pyrrolic nitrogen. As far as other nitrogen forms are concerned, the use of the Fe/Mo catalyst promotes the pyridinic configuration, while in the case of the Co/Mo catalyst, pyrrolic nitrogen is favored.

The inelastic mean free path of N 1s photoelectrons is about 1.8 nm at an excitation energy of 830 eV [33]. The information about the electronic state of nitrogen inside the MWCNTs is provided by NEXFAS spectroscopy in the TEY mode, which records photoelectrons from about 10 nm from the surface of the sample. NEXAFS N K-edge spectra of the studied materials exhibited three peaks before the σ* resonance (Figure 5b). The peaks at ≈398.5 and ≈400.0 eV are attributed to pyridinic and pyrrolic nitrogen, respectively [34–36]. An intense peak split at ≈401.0 eV corresponds to the vibrational fine structure of N_2_ molecules [37–38], which overlaps with the peak from graphitic nitrogen at ≈401.5 eV [39]. The height of this intense peak increases in the order of Ni < Co < Fe in accordance with the amount of N_2_ and graphitic nitrogen determined from the XPS N 1s spectra (Table S2 in Supporting Information File 1). Since the nitrogen molecules are mainly inside the nanotube, a consistency of the NEXAFS and XPS data indicates that most of the produced MWCNTs have thin walls consisting of about five layers.

### Raman scattering

**Figure 6:**
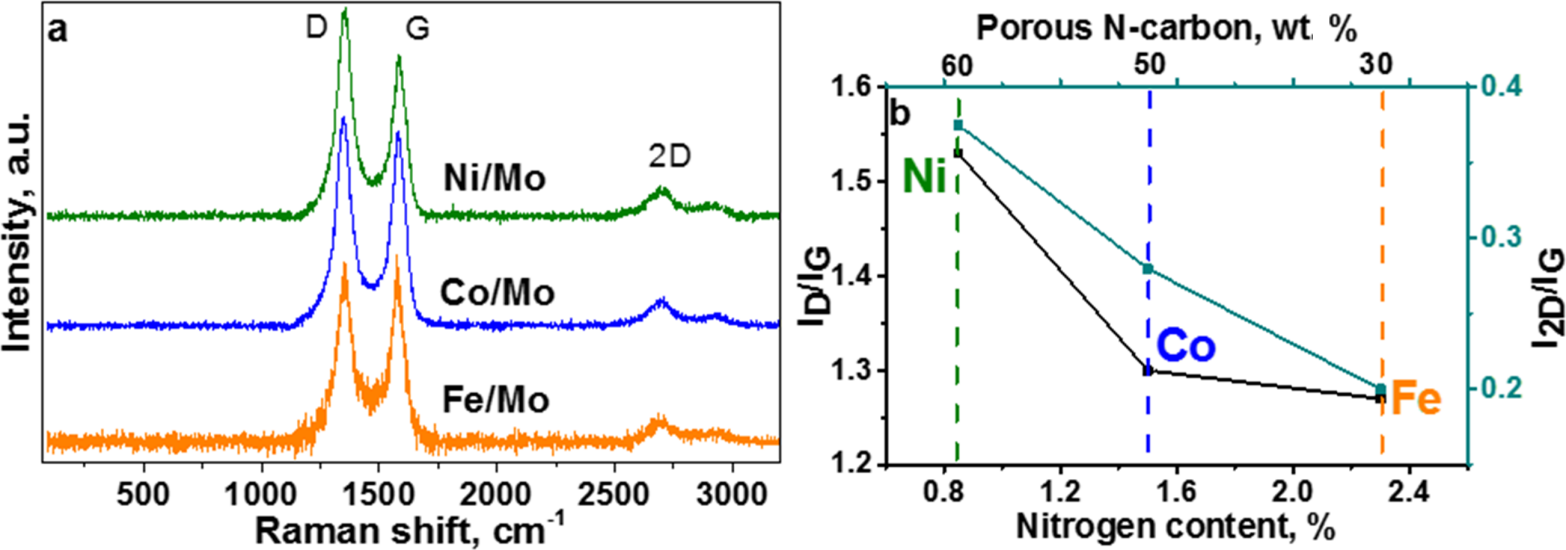
Raman spectra of CN*_x_* materials synthesized using Fe/Mo, Co/Mo, and Ni/Mo catalysts (a). Relationship between the ratios of Raman peak intensities and total nitrogen content in CN*_x_* materials (b).

In Raman scattering from 1000 to 2000 cm^−1^, the spectra of the samples exhibited D and G bands at ≈1350 and ≈1580 cm^−1^ (Figure 6a). The ratio of intensities of these bands is often used for the estimation of the disorder in graphitic materials [40]. The calculated *I*_D_/*I*_G_ value is equal to 1.27, 1.30, and 1.54 for the materials synthesized using Fe/Mo, Co/Mo, and Ni/Mo (Figure 6b). An increase in the *I*_D_/*I*_G_ ratio correlates with an increasing fraction of porous carbon in CN*_x_* materials (Table S1 in Supporting Information File 1). The substitution of carbon atoms by nitrogen also introduces disordering in the graphene lattice. It has been shown that this type of defect influences the 2D band intensity more than the D band [41]. There are many research works devoted to this issue and the common trend is a decrease of the *I*_2D_/*I*_G_ ratio with the amount of nitrogen incorporated in a CN*_x_* structure [41–44]. For our materials, the *I*_2D_/*I*_G_ ratio decreases almost linearly with the nitrogen content, while the *I*_D_/*I*_G_ ratio has no such obvious dependence (Figure 6b). Based on these data we conclude that the nitrogen defects strongly affect the 2D band, while the D band intensity in the Raman spectra of the materials under study is mainly influenced by topological defects in the porous carbon. The presence of the largest amount of structural defects in the material synthesized using Ni/Mo catalyst is supported by the analysis of XPS C 1s spectra. The fitting of the spectra has determined the highest intensity of the component for this material at around 285.3 eV, which arises from carbon atoms located in defective regions of graphitic material [45].

### Supercapacitor performance

**Figure 7:**
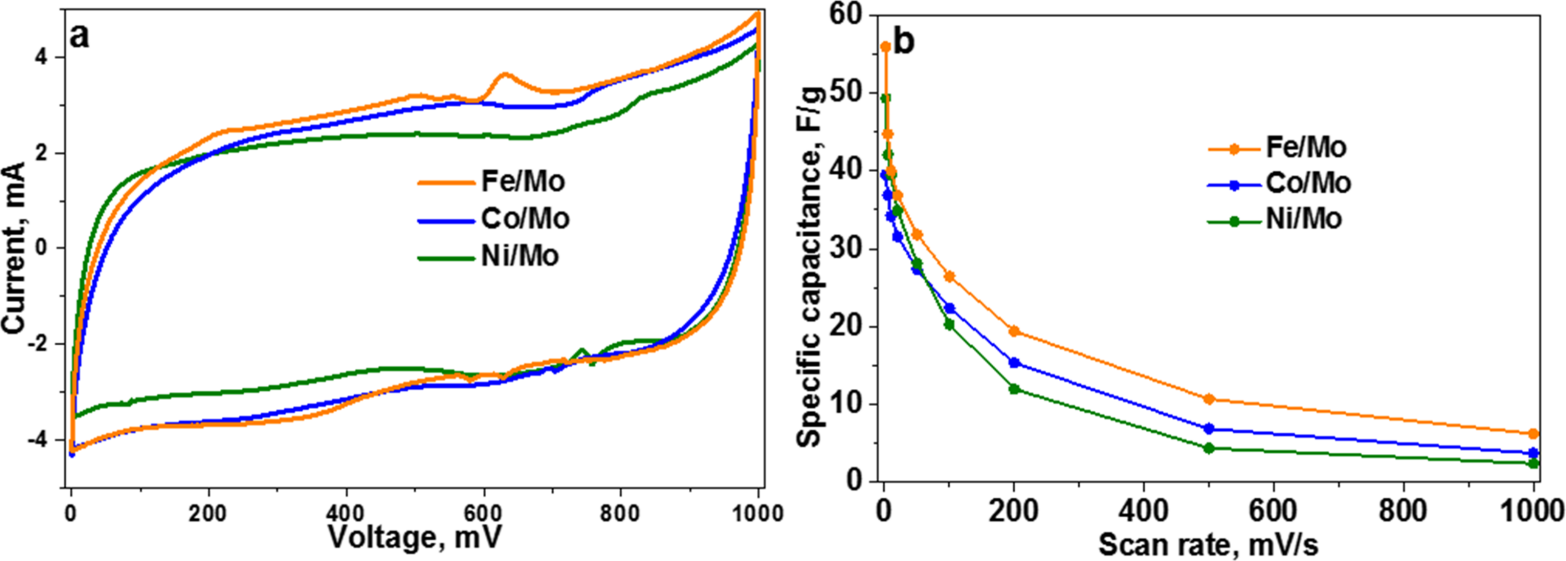
CV curves of CN*_x_* materials, synthesized using Fe/Mo, Co/Mo, and Ni/Mo catalysts, measured at a scan rate of 20 mV s^−1^ (a). Dependence of the specific capacitance of the CN*_x_* materials with the scan rate (b).

The CV curves of CN*_x_* materials recorded at a scan rate of 20 mV s^−1^ are compared in Figure 7a. The curves have a near rectangular shape, indicating a good capacitive behavior of the materials. The peaks and humps on the curves correspond to redox reactions with a participation of oxygen-containing groups and nitrogen species. In the used acidic electrolyte the electroactive groups are carbonyls [46] and pyridinic nitrogen [47]. Both of these functional groups are present in the CN*_x_* materials, as seen from the fitting of the XPS C 1s spectra (Figure S6 in Supporting Information File 1) and N 1s spectra (Figure 5a). Redox transformations of carbonyl groups and pyridinic nitrogen are observed at charge/discharge potentials of 260/100 mV [48] and 825/175 mV [47], respectively. Additionally, the CV curve of CN*_x_* material synthesized using the Fe/Mo catalyst shows a pronounced peak at ≈630 mV on the charging curve and its couple at ≈345 mV on the discharging curve (Figure 7a). These peaks are likely to be related to the redox activity of iron encapsulated in the MWCNTs [49]. Oxides and hydroxides of Co and Ni are not electrochemically active in an acidic environment [50], while redox transformations between Mo(VI) and Mo(IV) may appear at voltages of 90 mV on the charging curve and 220 mV on the discharging curve for Ag/AgCl reference electrode [51]. However, these peaks were not detected in the CV curves of our samples. The lower the scan rate of the applied potential, the larger the contribution of redox reactions to the capacitance. An increase in the specific capacitance determined at 2 mV s^−1^ in the order of the used catalyst as Co < Ni < Fe fully correlates with the total amount of oxygen and nitrogen in these materials (Table 1).

**Table 1:** Total nitrogen and oxygen content (atom %) determined from XPS data, BET specific surface area (m^2^ g^−1^), fraction of porous carbon evaluated from DTG analysis, and specific capacitance (F g^−1^) of CN*_x_* materials synthesized using different catalysts.

Catalyst	Nitrogen	Oxygen	*S*_BET_	Porous carbon	Specific capacitance	
					at 2 mV s^−1^	at 1000 mV s^−1^

Ni/Mo	0.9	5.0	207	0.6	50	2
Co/Mo	1.5	2.0	280	0.5	40	4
Fe/Mo	2.3	7.4	306	0.3	56	6

The specific capacitance of CN*_x_* materials decreases with the increasing the scan rate of the potential (Figure 7b) because the ions have no time for diffusion inside the material and can only easily adsorb on the available surface of the electrode. Hence, the capacitance at a high scan rate is mainly determined by the surface area of the electrode material. The largest and smallest specific capacitances were observed for the CN*_x_* materials synthesized using Fe/Mo and Ni/Mo catalysts and these materials possessed respectively the highest and the lowest specific surface area (Table 1). Note that the surface area for these samples does not correlate with the fraction of porous carbon. The sample with the lowest specific surface area, which was produced using the Ni/Mo catalyst, contains the largest amount of porous carbon, while the opposite behavior is observed for the sample synthesized using the Fe/Mo catalyst (Table 1). The reason is likely due to the different average number of layers in these porous carbons. High-resolution TEM images demonstrate a larger thickness of the former sample as compared to the latter one (Figure S2 in Supporting Information File 1). Despite low values of nitrogen content and low surface area, the sample synthesized using the Ni/Mo catalyst exhibited a good capacitive behavior at scan rates below 100 mV/s (Figure 7b), which confirms a benefit of the addition of porous carbon for the development of effective carbon-based supercapacitors.

### Impedance spectroscopy

**Figure 8:**
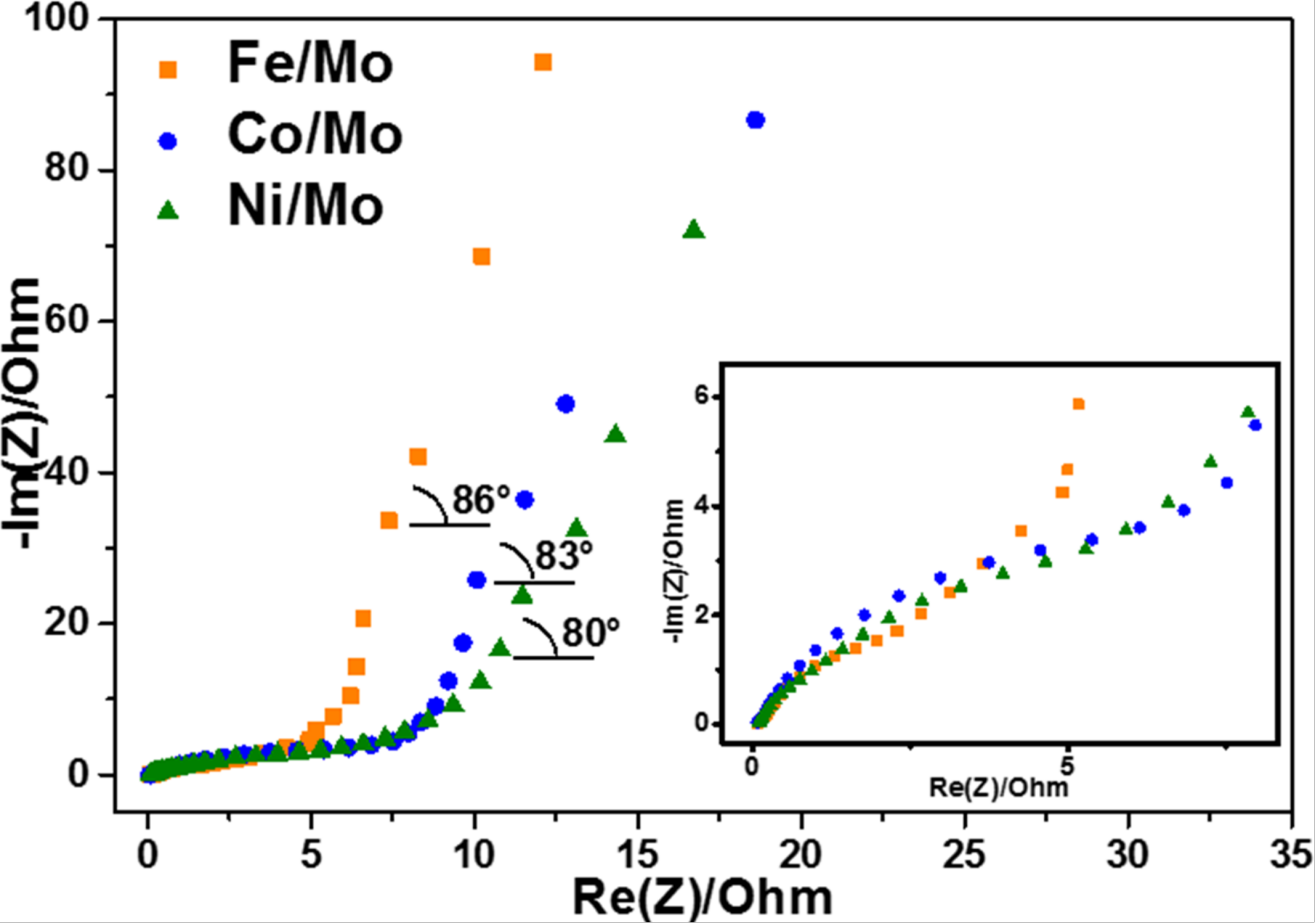
Electrochemical impedance spectroscopy (EIS) Nyquist plots of electrodes made from CN*_x_* materials synthesized using Fe/Mo, Co/Mo, and Ni/Mo catalysts. Inset shows a magnification of the high-frequency range.

The kinetics of the charge–discharge processes of the electrodes was investigated using EIS measurements. The impedance diagrams represented as Nyquist plots are shown in Figure 8. The Nyquist plot of an ideal supercapacitor is presented by a semicircle with a vertical line at low frequencies. The circle radius, *R*_ct_, characterizes the charge-transfer resistance, which is inversely proportional to the rate of electron transfer. The initial point of the circle at high frequency, *R*_f_, corresponds to the solid–solution resistance. The vertical line is the Warburg impedance, which arises from limitations of the ion diffusion in the electrolyte [52]. The values determined from the Nyquist plot analysis are collected in Table 2. The *R*_f_ values are close for all studied samples because they possess a good wettability due to the presence of oxygen-containing groups and incorporated nitrogen. The charge-transfer resistance *R*_ct_ (behavior is shown in inset in Figure 8) of electrodes increases with the nature of the catalyst according to Fe < Co < Ni in agreement with the decrease in nitrogen content in the materials (Table 1). Hence, the incorporation of nitrogen in the carbon network improves the electrical conductivity of the material. Similar results have been reported in [53], where the *R*_ct_ value increased as the nitrogen content in the carbon material decreased. The slope angle of the Warburg impedance is between 80° and 86° (Table 2), which indicates a high rate capability of the CN*_x_* electrodes. The value is larger when the fraction of porous carbon in the sample is lower (Table S1 in Supporting Information File 1). This fact evidences the role played by the MWCNTs in the enhancement of the charge transport properties of the materials. The fast ion-transfer process in the cells results in high power densities of supercapacitors, which are equal to 34.6 kW/kg, 29.4 kW/kg, and 20 kW/kg for the CN*_x_* materials produced using Fe/Mo, Co/Mo, and Ni/Mo catalysts, respectively. These values are substantially higher than the best power density of 10 kW/kg found for supercapacitors with activated carbon [2].

**Table 2:** Fitting parameters of electrochemical impedance spectra of CN*_x_* materials synthesized using different catalysts.

Catalyst	*R*_f_ (Ω)	*R*_ct_ (Ω)	Angle of tail^a^ (°)

Fe/Mo	0.09	9	86
Co/Mo	0.07	11	83
Ni/Mo	0.09	16	80

^a^The slope angle of the Warburg impedance.

## Conclusion

Nitrogen-doped porous carbon/MWCNT hybrid materials were synthesized using cluster molecules containing molybdenum and transition metal as a source of catalytic nanoparticles for the CCVD synthesis. The cluster molecules were distributed over a MgO support before oxidation in air at 700 °C. The catalysts were activated in a CH_3_CN/CH_4_/H_2_ flow with a slow heating ramp from room temperature to 900 °C. This resulted in the growth of MWCNTs on in situ generated metal catalytic nanoparticles, and formation of porous carbon over the MgO support. TGA analysis of the materials evidenced the presence of three components, which were attributed to porous carbon, highly defective MWCNTs and more perfect MWCNTs. The ratio of these fractions as well as the concentration of the incorporated nitrogen were shown to depend on the nature of the metal catalyst. The material synthesized using the Fe/Mo catalyst contained the largest amount of nitrogen and the smallest proportion of porous carbon. Such a correlation could indicate that in the used synthetic conditions, the nitrogen atoms are mainly incorporated within the walls of the MWCNTs. The CN*_x_* hybrids were investigated as electrode materials for supercapacitors and showed a good power density in a 1 M H_2_SO_4_ electrolyte. It is shown that the power density is improved with an increase in the fraction of MWCNTs and the porous carbon provides good capacitance for the electrode, while nitrogen atoms were found to decrease the charge-transfer resistance of the cell. A balance between the different structural parameters of the CN*_x_* hybrid allows for electrode materials of great potential for use in energy storage applications.

## Supporting Information

File 1 Additional experimental information.
SEM images of CN*_x_* samples; High-resolution TEM images of porous carbon interlinking with CNTs; TEM images with histograms of diameter distribution of CNTs; NEXAFS C K-edge spectra of CN*_x_* samples; TG analysis of thermal oxidation of CN*_x_* materials and comparison of the DTG curves; the ratios of different structural components in CN*_x_* materials; total nitrogen concentration and percentages of various nitrogen forms in CN*_x_* materials; the XPS C 1s spectra of CN*_x_* samples.
