# Supplementary material for: One-step chemical vapor deposition synthesis and supercapacitor performance of nitrogen-doped porous carbon–carbon nanotube hybrids
Source: Beilstein J Nanotechnol. 2017 Dec 12;8:2669–79. doi: 10.3762/bjnano.8.267 (PMC5753048; doi:10.3762/bjnano.8.267)
Supplement: File 1 — Additional experimental information. SEM images of CNx samples; High-resolution TEM images of porous carbon interlinking with CNTs; TEM images with histograms of diameter distribution of CNTs; NEXAFS C K-edge spectra of CNx samples; TG analysis of thermal oxidation of CNx materials and comparison of the DTG curves; the ratios of different structural components in CNx materials; total nitrogen concentration and percentages of various nitrogen forms in CNx materials; the XPS C 1s spectra of CNx samples. [file Beilstein_J_Nanotechnol-08-2669-s001.pdf]

Supporting Information

for

One-step chemical vapor deposition synthesis and supercapacitor performance of nitrogen-doped porous carbon–carbon nanotube hybrids

Egor V. Lobiak*[1], Lyubov G. Bulusheva[1,2], Ekaterina O. Fedorovskaya[1,2], Yury V. Shubin[1,2], Pavel E. Plyusnin[1,2], Pierre Lonchambon[3], Boris V. Senkovskiy[4,5], Zinfer R. Ismagilov[6,7], Emmanuel Flahaut[3], and Alexander V. Okotrub[1,2]

Address: [1]Nikolaev Institute of Inorganic Chemistry, SB RAS, 630090 Novosibirsk, Russia, [2]Novosibirsk State University, 630090 Novosibirsk, Russia, [3]CNRS, Institut Carnot Cirimat, F-31062 Toulouse, France, [4]St. Petersburg State University, 7-9, Universitetskaya Nab., St. Petersburg 199034, Russia, [5]II Physikalisches Institut, Universität zu Köln, Zülpicher Straße 77, 50937 Köln, Germany, [6]Boreskov Institute of Catalysis, SB RAS, 630090 Novosibirsk, Russia, and [7]Institute of Coal Chemistry and Materials Science FRC CCC SB RAS, Kemerovo 650000, Russia

Email: Egor V. Lobiak - lobiakev@niic.sbras.ru

* Corresponding author

Additional Experimental Information

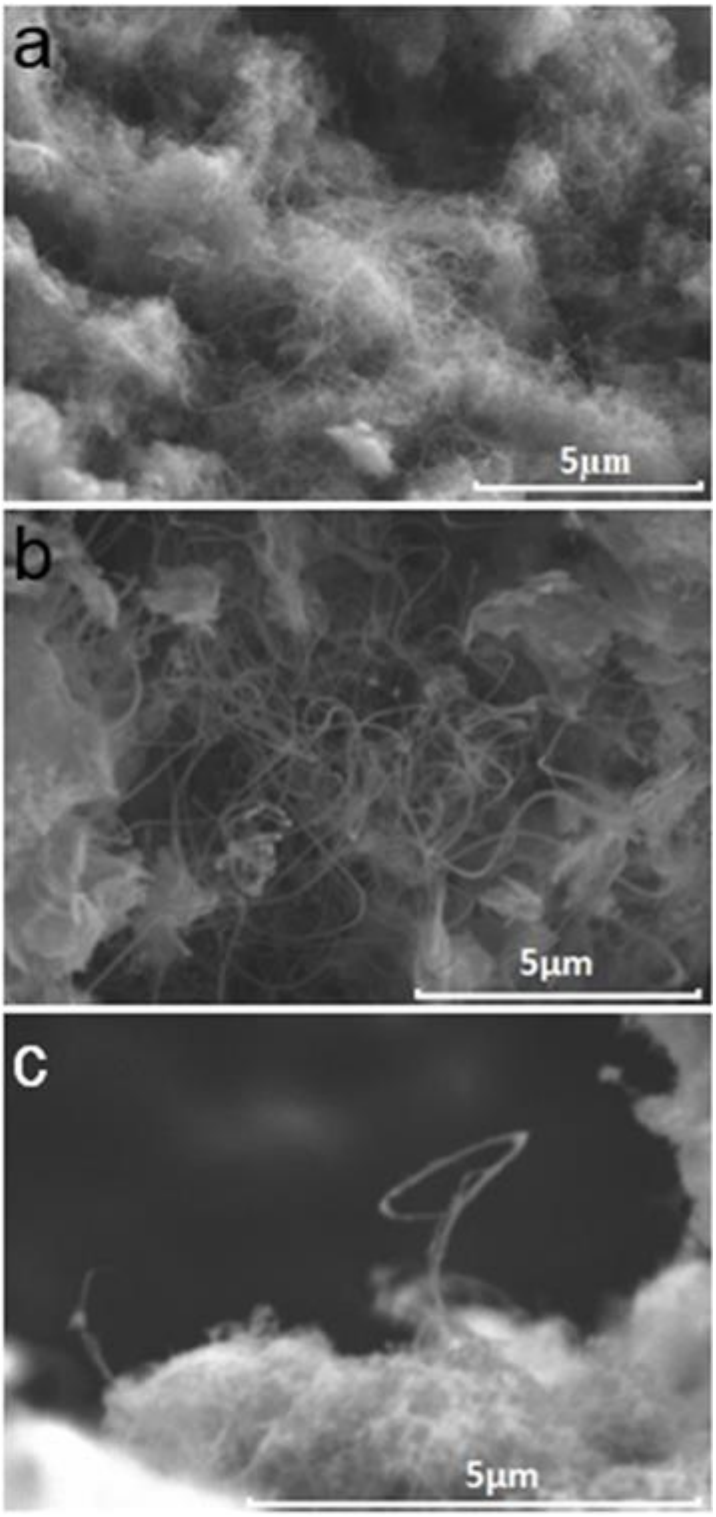

Figure S1: SEM images of CN_x samples synthesized using (a) Co/Mo, (b) Ni/Mo and (c) Fe/Mo catalysts. The samples were purified from the MgO support and the images demonstrate the co-existence of CNTs and non-tubular carbon.

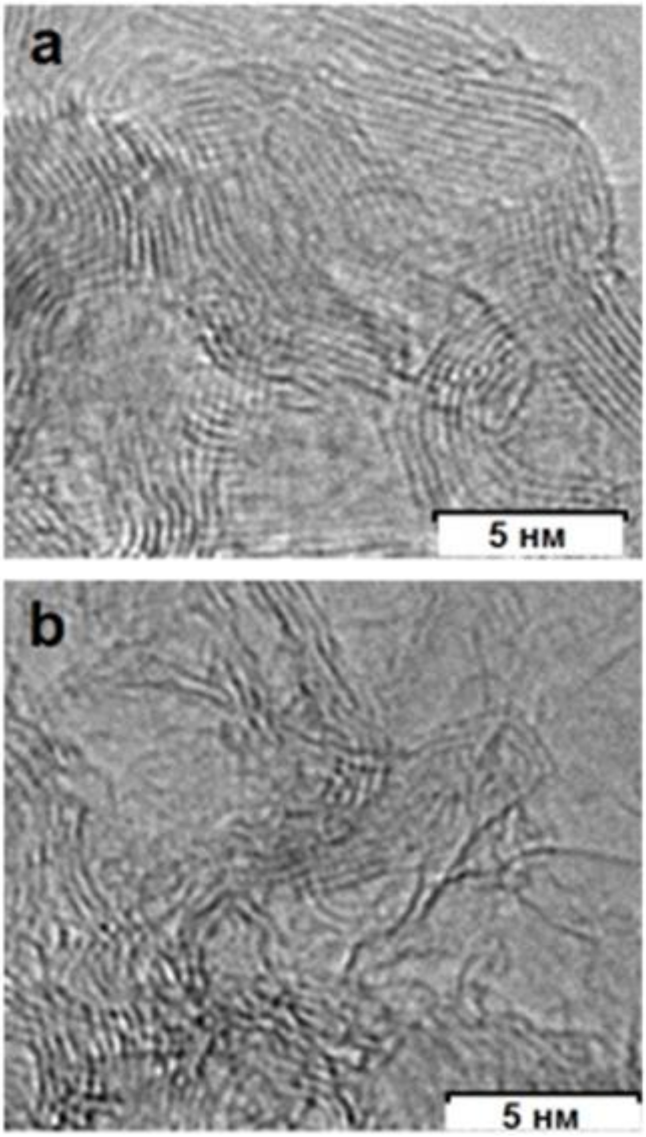

Figure S2: High resolution TEM images of porous carbon interlinking with CNTs synthesized using (a) Ni/Mo, (b) Fe/Mo catalysts.

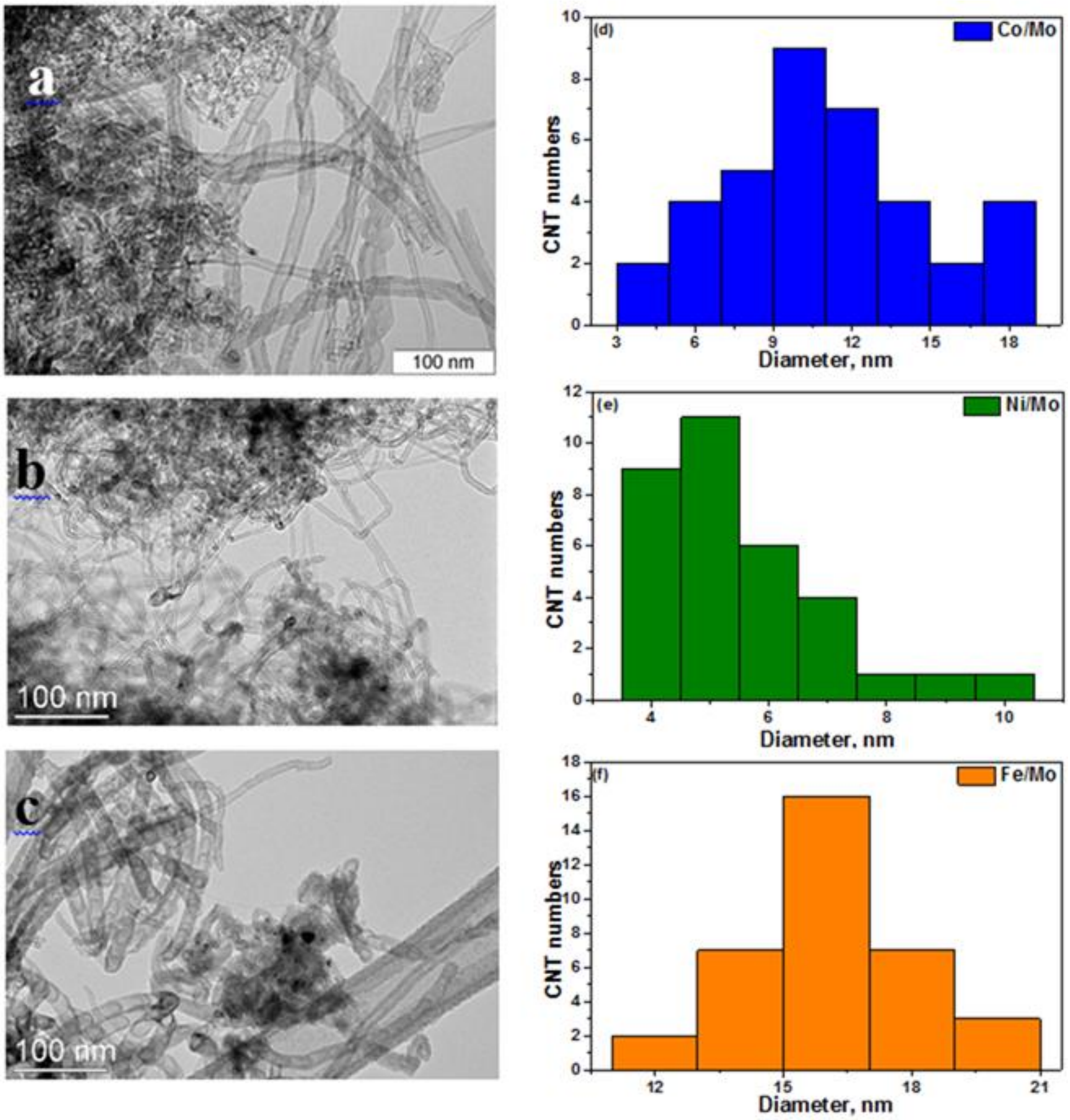

Figure S3: TEM images of CN_x samples synthesized using (a) Co/Mo, (b) Ni/Mo and (c) Fe/Mo catalysts, which have been used for evaluation of the outer diameter distribution of CNTs. Histograms of diameter distribution of CNTs are presented in (d) Co/Mo, (e) Ni/Mo and (f) Fe/Mo.

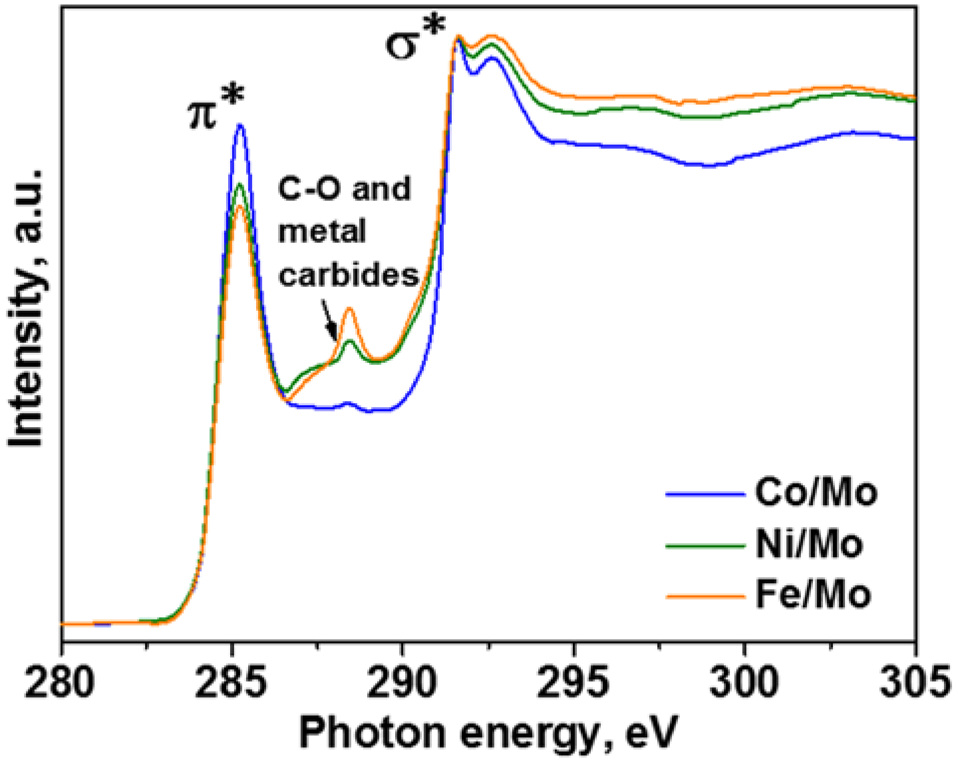

Figure S4. NEXAFS CK-edge spectra of CN_x samples synthesized using Co/Mo, Ni/Mo and Fe/Mo catalysts.

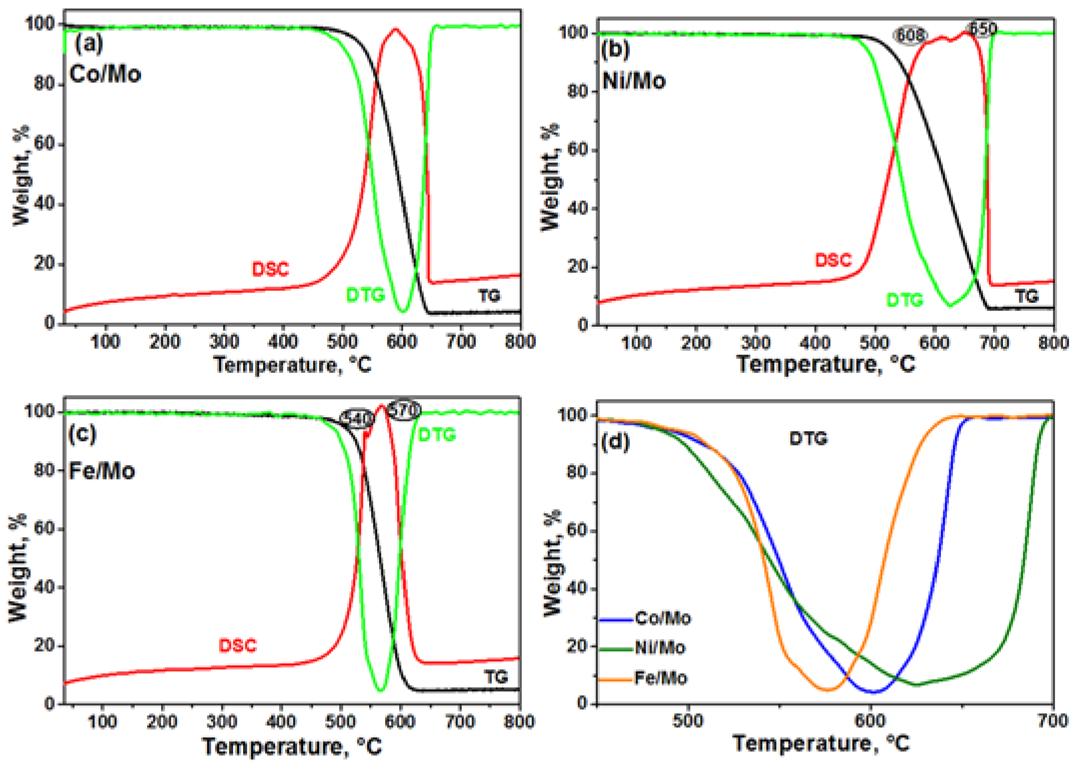

Figure S5: TG, DTG and DSC curves of thermal oxidation of CN_x materials synthesized using (a) Co/Mo, (b) Ni/Mo, (c) Fe/Mo catalysts. (d) Comparison of DTG curves of CN_x materials.

Table S1. Ratios of integral intensities of components in the DTG curves of CN_x materials synthesized using different catalysts and the peak temperature of each component.

Catalyst	Component 1	Component 2	Component 3
Co/Mo	0.5 (566 °C)	0.3 (602 °C)	0.2 (627 °C)
Ni/Mo	0.6 (580 °C)	0.2 (640 °C)	0.2 (670 °C)
Fe/Mo	0.3 (539 °C)	0.45 (566 °C)	0.25 (591 °C)

Table S2. Total nitrogen concentration (at.%) and percentages of various nitrogen forms in CN_x materials synthesized using different catalysts. The values were obtained from the XPS data.

Catalyst	N total, at.%	N pyridinic	N pyrrolic	N graphitic	N oxidized	N_2
Ni/Mo	0.9	24.4%	22.4%	35.6%	9.2%	8.3%
Co/Mo	1.5	19.2%	25.4%	39.5%	6.5%	9.4%
Fe/Mo	2.3	21.4%	13.5%	45.0%	6.7%	13.4%

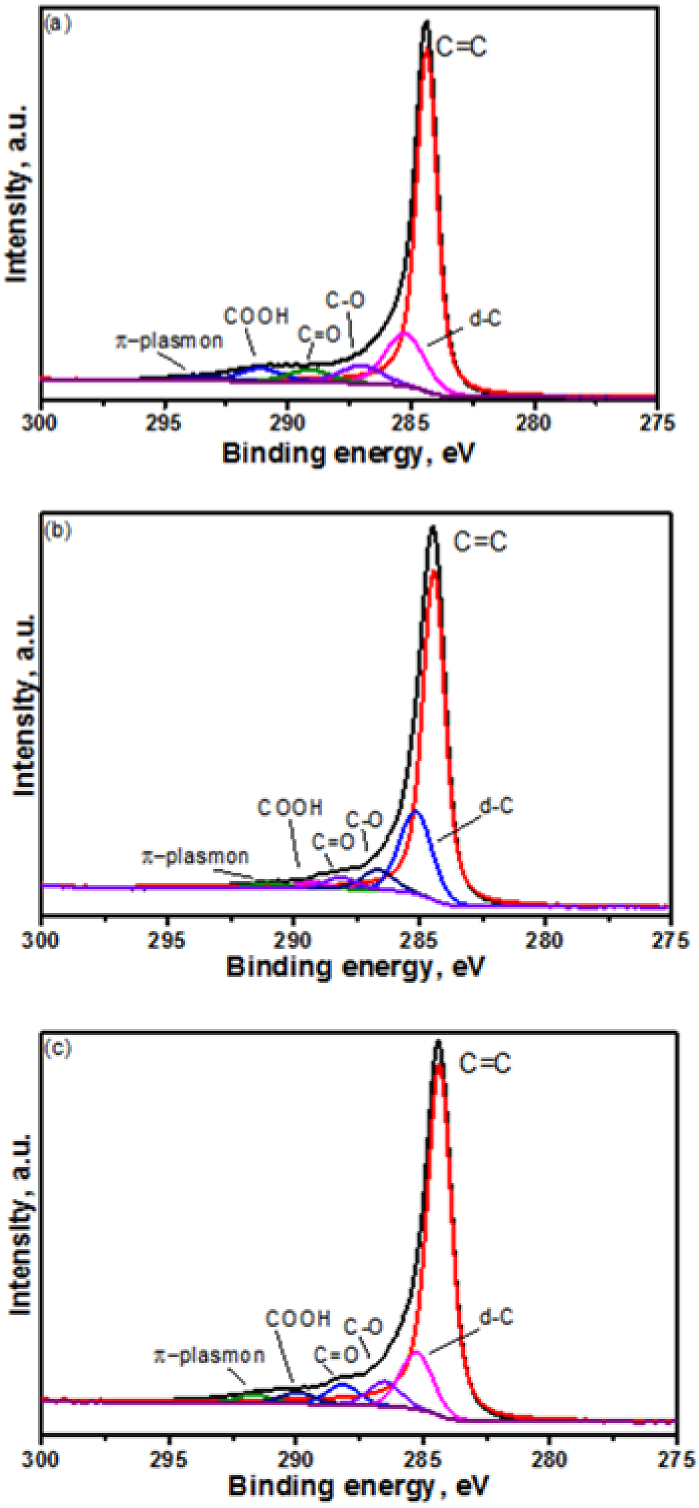

Figure S6: XPS C1s spectra of CN_x materials synthesized using (a) Co/Mo, (b) Ni/Mo and (c) Fe/Mo catalysts.
